# Mitochondrial oxidative stress promotes the accumulation of advanced glycation end products

**DOI:** 10.1371/journal.pone.0352355

**Published:** 2026-06-23

**Authors:** Firoz Akhter, Sourav Samanta, Alexandre A. Sosunov, Huawei Wu, Ethan Kim Tieu, Shi Fang Yan, Shirley ShiDu Yan

**Affiliations:** Division of Surgical Science of Department of Surgery, Columbia University in New York, New York, United States of America; School of Life Sciences, H.N.B. Garhwal University (A Central University), INDIA

## Abstract

Advanced glycation end products (AGEs) are a class of toxic metabolites that contribute to disease progression. In our previous study, we demonstrated that age-related AGE accumulation is associated with mitochondrial dysfunction. However, the direct link between mitochondrial dysfunction and AGE accumulation within the context of AD pathogenesis has not yet been fully explored. It also remains unclear whether mitochondrial stress and mitochondrial reactive oxygen species (ROS) drive the accumulation of AGEs. This study, for the first time, provides evidence of progressive AGE accumulation in the cortical mitochondria of AD mice exhibiting mitochondrial dysfunction and Aβ pathology. AGE levels were significantly correlated with Aβ-induced mitochondrial dysfunction, oxidative stress, and amyloid pathology. Notably, mitochondrial stress induced by a mitotoxin significantly increased the accumulation of AGEs in cellular and mitochondrial compartments. Scavenging mitochondrial ROS using the mitochondria-targeted antioxidant reduced AGE accumulation and improved mitochondrial function. Our findings highlight the role of mitochondrial dysfunction in AGE metabolism and provide new insights into the pathogenesis of AD.

## Introduction

Alzheimer’s disease (AD) is a major contributor to dementia in older adults, characterized by cognitive decline and the accumulation of the β-amyloid peptide (Aβ) in the brain [1]. The disease arises from complex multifactorial mechanisms, including mitochondrial dysfunction, protein aggregation, and oxidative stress [2,3]. The occurrence of AD is significantly higher in an aging population, as well as in subjects with dysregulation in glucose metabolism, which could be one of the causal factors for AD. The age-related accumulation of toxic metabolites such as advanced glycation end products (AGEs), is of significant clinical important due to their association with oxidative stress and neuronal dysfunction, both of which contribute to the pathogenesis of chronic diseases such as AD [4–10]. AGE levels increase during biological aging, largely due to a decline in the efficiency of homeostatic processes. AGEs are closely linked to AD pathology and are thought to contribute to the formation of amyloid pathology and neurofibrillary tangles [8,9,11]. In addition, Aβ promotes neuronal perturbation through mechanisms involving reactive oxygen species (ROS) and AGEs [12–17]. Progressive glycation further impairs mitochondrial energy production and exacerbates oxidative stress [5–7,18]. However, the underlying causes and consequences of AGE accumulation in AD or Aβ-rich brains remain to be fully explored.

Mitochondrial dysfunction is a key pathological feature of AD. Increasing evidence from multiple independent studies demonstrates the presence of AD- or Aβ-associated mitochondrial dysfunction in the brain of AD patients and in various Aβ/AD mouse models [13,14, 19–43]. Impairments in mitochondrial bioenergetics, along with excessive production of ROS, have been widely observed in Aβ/AD brains. Our previous studies have shown that age-related accumulation of harmful metabolites, such as AGEs, is associated with mitochondrial stress and oxidative stress [6,7]. However, the relationship between the accumulation of AGEs and mitochondrial dysfunction during disease progression remains poorly understood.

In the present study, we investigate the mechanisms underlying the accumulation of AGEs in brain and mitochondria of AD mice exhibiting mitochondrial dysfunction and Aβ pathology, as well as their relevance to AD pathogenesis. Specifically, we address several key unanswered questions: Do AGEs accumulate in the cerebral mitochondria of Aβ-rich brain? Is AGE accumulation correlated with Aβ pathology and mitochondrial dysfunction? Is AGE accumulation associated with mitochondrial oxidative stress in an Aβ-enriched environment? Do dysfunctional mitochondria and mitochondrial oxidative stress contribute to AGEs buildup? If so, can inhibition of mitochondrial ROS reduce AGEs formation/accumulation and restore mitochondrial function? To address these questions, we comprehensively analyze AGEs levels, ROS production, and mitochondrial function in a mouse model of AD with amyloid pathology. In addition, we examine the impact of mitochondrial ROS on AGE accumulation and mitochondrial function in human neuronal cell lines. Overall, our study explores the link between mitochondrial perturbation and the accumulation of toxic metabolites, such as AGEs, in the context of amyloid pathology.

## Materials and methods

### Animal study

Both male and female transgenic AD mice and non-transgenic mice (nonTg), aged 4–12 months, were used in this study. Transgenic AD mice (referred to as mAPP in this text) were obtained from Jackson Laboratory (MMRRC Strain# 034836-Jax, J20 line) [44] and express a mutant form of the human amyloid protein precursor bearing both the Swedish (K670N/M671L) and the Indiana (V717F) mutations (APPSwInd), along with human Aβ. mAPP mice were initially purchased from The Jackson Laboratory and subsequently bred and housed for multiple generations in our institution's animal facility. Housing conditions were maintained under controlled status, with constant environmental parameters, including a temperature of 21 ± 1°C, a 12-hour light-dark cycle, and a pathogen-free environment. The mice were fully acclimated to the laboratory conditions. Mice were decapitated without anesthesia to avoid potential effects of anesthetics on neuronal and mitochondrial function. Brain tissues were then collected, snap-frozen in liquid nitrogen prior to storage at −80ºC for subsequent analysis.

All animal studies were conducted in accordance with the National Institutes of Health guidelines for the care and use of laboratory animals and were approved by the Institutional Animal Care and Use Committee at Columbia University (AC-AABR8628).

### MG-AGEs preparation *in vitro*

BSA glycation was performed following our previously described method [5]. Briefly, native BSA (Sigma, A7030) was dissolved in phosphate buffered saline (PBS) and incubated with 100 mM methylglyoxal (MG) (Sigma, 67028) at 37ºC for 12 days. Glycated BSA (MG-BSA) on day 12 was then dialyzed against PBS and filtered through a 0.22 µm membrane.

### Mitochondrial isolation

Mitochondria were isolated from mouse cortices as our previous described [15]. Cortical homogenates were centrifuged at 1,300 g for 5 min, and the supernatant was adjusted to 15% Percoll and centrifuged at 34,000 g for 10 min. The mitochondrial pellet was resuspended in isolation buffer (225 mM D-mannitol, 75 mM sucrose, 2 mM K_2_HPO_4_, 5 mM HEPES, pH 7.2) with 0.02% digitonin, centrifuged at 8,000 g for 10 min, washed twice, and re-centrifuged. Protein concentration was measured using the Bio-Rad DC assay.

### Immunoblot analysis

Cortical brain homogenates were subjected to immunoblotting with the following primary antibodies: Rabbit anti-Lamp-1 (Santa Cruz Biotechnology, sc-17768, 1:5000,), calnexin (Cell Signaling Technology, 2433S, 1:5000,), synaptophysin (Cell Signaling Technology, 36406S (D8F6H), 1:5000), VDAC (Calbiochem, 185−197, 1:5000,), TOM20 (Santa Cruz Biotechnology, SC-17764, 1:5000,), and mouse anti-β actin (Sigma, A5441, 1:5000). HRP-conjugated anti-rabbit or anti-mouse IgG antibody (Sigma, A6154 or A4416, 1:5000–10,000) was used as the corresponding secondary antibody. Protein signals were visualized using SuperSignal™ West Pico PLUS Chemiluminescent Substrate (Thermo Fisher, 34578) and a a FluorChem HD2 imaging system.

### Immunodetection of AGEs and Aβ

Immunodot blot was used to assess the distribution and levels of AGE-modified proteins in each experimental group, as described previously. Briefly, equal amounts of total protein (2 µg) from mouse cortical lysates at 4 and 12 months were spotted onto nitrocellulose membranes (Bio-Rad). β-actin (Sigma, A5441) was used as a loading control. Primary antibodies against methylglyoxal AGEs (MG-AGEs) (Cell Biolabs, STA 011) and carboxymethyl lysine AGEs (CML-AGEs) (Cosmo Bio, KAL-KH024) were applied at 1:3000, followed by HRP-conjugated secondary antibodies (1:5000). Signals were detected using chemiluminescent substrate (Thermo Fisher, 34580) and visualized with a FluorChem HD2 system. Signal intensity was quantified using ImageJ (NIH, Bethesda, MD, USA). For ELISA, MG-AGEs and CML-AGEs in mouse cortical lysates were measured using commercial kits (My BioSource, MBS756134 and MBS265592) following the manufacturer’s instructions.

***Aβ measurement.*** Brains from mAPP and nonTg mice were extracted with 5.0 M guanidine-50 mM Tris-HCl, pH8.0) to quantified Aβ40 and Aβ42 levels in brain tissue [15].Total human Aβ40 and Aβ42 levels were measured using ELISA kit (Invitrogen/Thermo Fisher Scientiﬁc, KHB3481 and KHB3441) following the manufacturer’s instructions.

### Measurement of mitochondrial respiratory chain enzyme activities and ATP

Activities of key mitochondrial respiratory chain enzymes, including complex I (NADH: ubiquinone oxidoreductase) and complex IV (cytochrome c oxidase), were measured in brain samples as described previously [16,45]. For complex I, protein was added to reaction buffer (5 mM MgCl₂, 2 mM KCN, 0.13 mM NADH, 2 µg/mL Antimycin) and the reaction initiated with Coenzyme Q1 (65 µM). After 180 s, Rotenone (2 µg/mL) was added, and NADH oxidation was monitored at 340 nm every 20 s for 18 readings. For complex IV, protein was added to assay buffer (10 mM Tris–HCl, pH 7.0, 120 mM KCl) and adjusted with dilution buffer (10 mM Tris–HCl, pH 7.0, 250 mM sucrose). The reaction was started with 25 µL ferrocytochrome c (0.22 mM), and cytochrome c oxidation was measured at 550 nm every 10 s for 6 readings.

ATP levels were measured using an ATP Bioluminescence Assay Kit (Roche) according to the manufacturer’s instructions [46]. Brain tissues were homogenized in the provided lysis buffer, incubated on ice for 30 min, and centrifuged at 12,000 × g for 10 min. ATP in the supernatant was quantified using a luminescence plate reader (Molecular Devices) with a 1.6 s delay and 10 s integration time.

### Mitochondrial ROS and antioxidant measurements

Mitochondrial ROS was assessed in situ using MitoSOX fluorescence in AGEs (100 µg/ml) or vehicle treated SK-N-SH cells (ATCC HTB-11) with or without 2 µM MitoTEMPO (2-(2,2,6,6-Tetramethylpiperidin-1-oxyl-4-ylamino)-2-oxoethyl) triphenylphosphonium chloride, Sigma SML0737, mTEMPO) for 24 h. Cells were incubated with 2.5 µM MitoSOX (Thermo Fisher, M36008) for 30 min, fixed, and imaged using a confocal microscope (Leica TCS SPE).

For ex vivo brain analysis, mice were sacrificed by cervical dislocation, and brains were extracted and sliced (~300 µm) using a tissue chopper (Electron Microscopy Sciences). Slices were perfused in an interface chamber (Harvard Apparatus) at 29 °C with ACSF (124 mM NaCl, 4.4 mM KCl, 1 mM Na₂HPO₄, 25 mM NaHCO₃, 2 mM CaCl₂, 2 mM MgCl₂, 10 mM glucose) continuously bubbled with 95% O₂/5% CO₂. After 1.5 h, slices were stained with 1 µM MitoSOX Red and DRAQ5 (1:500) for 30 min, washed with PBS, and imaged using a confocal microscope (Nikon AXR, 20 × objective).

ROS levels in brain lysates were measured by electron paramagnetic resonance (EPR) using a Bruker EMXnano spectrometer. Cortical tissues were incubated with 1-hydroxy-3-methoxycarbonyl −2,2,5,5-tetramethylpyrrolidine-HCl (CMH, 0.25 mM) spin probe for 1 h at 37 °C and lysed using Kimble^TM^ Kontes^TM^ Pellet Pastle^TM^ Cordless Motor (Fisher Scientific). Finally, 2 mg/ml protein lysate in 50 µl was drawn up into a glass EPR capillary (BLAUBRAND) and analyzed with a Bruker EMXnano EPR spectrometer. EPR peak height is defined as the vertical intensity between peaks in the derivative spectrum and is utilized in the quantitative analysis of EPR spectra to determine ROS levels.

Hydrogen peroxide (H_2_O_2_) levels in mouse cortical tissue were measured using the Amplex Red Hydrogen Peroxide/ Peroxidase Assay Kit (Invitrogen, A22188) following the manufacturer’s instructions. Briefly, 50 µg of protein from homogenized samples was used, and background signal from reactions without tissue was subtracted from the reading.

### Mitochondrial respiration

Real-time mitochondrial oxygen consumption rate (OCR) and extracellular acidification rate (ECAR) were measured using the Seahorse XFe96 Analyzer with the XF Cell Mito Stress Assay Kit (Agilent, 103015−100) as described previously [7]. Human neuron-like SK-N-SH cells (ATCC HTB-11) were cultured in low-glucose DMEM (Thermo Fisher, 11-885-092) with 10% FBS and 1% penicillin/streptomycin. Cells were seeded at 20,000 cells/well in XF96 plates and, after 24 h, media was replaced with serum-free DMEM. Cells were pretreated with 2 µM MitoTEMPO for 30 min, followed by 24 h incubation with 100 µg/mL AGEs with or without MitoTEMPO. Before the assay, cells were washed with Seahorse assay media (DMEM, pH 7.4, 10 mM glucose, 2 mM glutamine) and incubated in assay media containing AGEs or AGEs + MitoTEMPO at 37 °C (non-CO₂ incubator) for 1 h. OCR and ECAR were measured in response to oligomycin (2.5 µM), FCCP (2 µM), and rotenone/antimycin A (0.5 µM). Data were normalized to protein content per well and analyzed using the Seahorse Analyzer and GraphPad Prism 9.

### Immunocytochemistry

Neuronal cell line (SK-N-SH cell, ATCC HTB-11) was grown on coverslips in 24-well plates and treated with KCN (500 nM), Rotenone (10 nM), or AGEs (100 µg/mL) in the absence and presence of mitoTEMPO (2 µM) for 24 h. Cells were then washed with PBS and fixed with 4% paraformaldehyde for 30 min at room temperature. After fixation, cells were washed again with PBS, permeabilized with 0.1% Triton X-100, and blocked with 5% goat serum for 60 min. Cells were then incubated with primary antibodies, TOMM20 (Thermo Fisher, MA5–32148, 1:200) or AGE (Antibodies-online, ABIN738516, 1:250). Following several washes with PBS, cells were incubated with an appropriate fluorophore-conjugated secondary antibody for 1 hour in the dark. After final washes, nuclei were counterstained with DAPI, and coverslips were mounted onto slides using mounting medium. Stained cells were visualized and imaged using a confocal microscope (Nikon AXR) and subsequently analyzed using ImageJ and GraphPad.

### Immunogold electron microscope

Mice were fixed by intracardiac perfusion with cold 3% paraformaldehyde and 0.25% glutaraldehyde in PBS. Small pieces of cortex were additionally fixed overnight in the same fixative and embedded in LR White resin. Ultrathin sections (~300 nm) were blocked by 10% donkey serum (30 min, RT) and primary anti-AGE antibodies produced in our Lab [9] were applied at 1:50 overnight. Secondary antibodies conjugated with 12nm gold particles were applied for 1.5 h at RT. Sections without primary AGE antibody were used as control. Sections were contrasted with uranyl acetate and examined under electron microscope Joel. 1200.

### Statistical analysis

Statistical analyses were performed using unpaired t-tests or one- and two-way ANOVA, followed by Fisher’s LSD test, in GraphPad Prism 9. Data are presented as mean ± SEM, and p < 0.05 was considered statistically significant.

## Results

### AGEs correlate with cerebral Aβ burden in AD mouse brain

**Fig 1:**
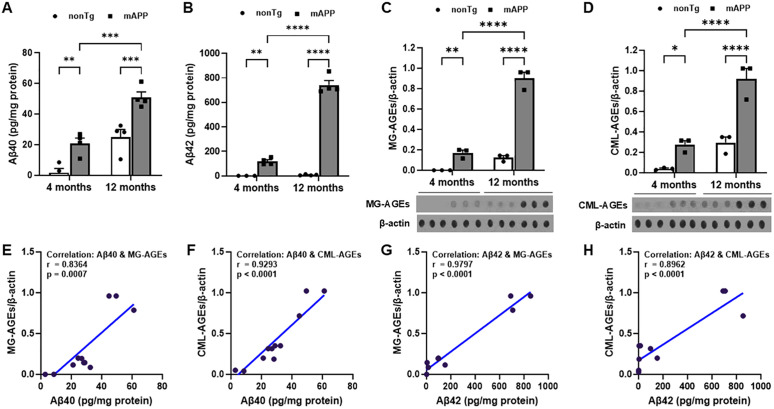
Accumulation of AGEs in cerebral cortex of mAPP mice. **(A-B)** Human Aβ40 and Aβ42 levels in the cerebral cortex of mAPP and nonTg mice at 4 and 12 months of age by ELISA specific for detection of human Aβ. **(C-D)** Quantification of MG-AGE and CML-AGE levels in the cerebral cortex of the mAPP and nonTg mice by immunodot blot, normalized to β-actin. Data were expressed as Mean ± SEM (n = 3-4 mice per group) and analyzed using 2-way ANOVA followed by the Fisher LSD test. *P < 0.05, **P < 0.01, ***P < 0.001, ****P < 0.0001. **(E-H)** Pearson correlation among cerebral MG-AGE and CML-AGE accumulation with the presence of cerebral Aβ40 and Aβ42.

Since mitochondrial dysfunction is a pathological feature of AD brains and Aβ can impair mitochondria, we investigated whether Aβ-mediated mitochondrial dysfunction influences AGE accumulation in Aβ-rich brains of AD mice. To this end, transgenic AD mice overexpressing human Aβ (mAPP mice) and exhibiting mitochondrial and amyloid pathology [16,17] were used. As shown in Fig 1A and 1B, cerebral Aβ levels progressively increased with age (Fig 1A and 1B). MG derived AGEs, a biologically significant species of AGEs, were significantly elevated in the cortex of mAPP mice compared to non-transgenic (nonTg) controls in an age-dependent manner. Accordingly, CML-AGEs, which are implicated in disease pathogenesis and cellular perturbation, were also increased in mAPP mice. Notably, the levels of AGEs were especially high at 12 months of age, a time of tremendous accumulation of cerebral Aβ, as compared to 4 months of age (Fig 1C and 1D). Pearson correlation analysis was performed to assess the potential relationship among MG-AGEs, CML-AGEs, Aβ40, and Aβ42 accumulation in the mouse brain. Both MG-AGEs and CML-AGEs positively correlated with Aβ40 and Aβ42 levels (Fig 1E-H), indicating that AGE accumulation progresses in the Aβ-rich environment.

### AGE accumulation in cortical mitochondria of Aβ-expressing AD mice

**Fig 2:**
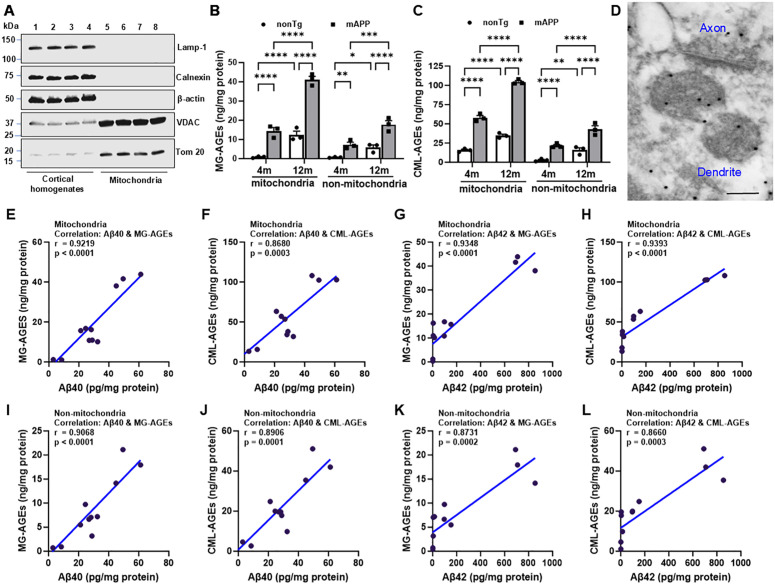
Increased accumulation of AGEs in cortical mitochondria of AD mice. **(A)** Immunoblotting of cortical homogenates and mitochondria fraction for lysosome (Lamp-1), endoplasmic reticular (ER, Calnexin), mitochondrial markers (VDAC and TOM20). **(B-C)** Age-related MG-AGE and CML-AGE accumulation in mitochondrial and non-mitochondrial fractions from nonTg and mAPP mice at 4 and 12 months of age by ELISA. N = 3 mice per group, data were analyzed using 2-way ANOVA followed by the Fisher LSD test. *P < 0.05, **P < 0.01, ***P < 0.001, ****P < 0.0001. **(D)** Immunogold EM images with a specific AGE antibody, followed by a gold-conjugated antibody (12 nm), demonstrating the presence of intramitochondrial AGEs accumulation (black particles) in 8-month-old mAPP mice. Scale bars = 150 nm. **(E-H)** Pearson correlations between mitochondrial MG-AGE and CML-AGE accumulation and cerebral Aβ40 and Aβ42 levels. **(I-L)** Pearson correlations between non-mitochondrial MG-AGE and CML-AGE accumulation and cerebral Aβ40 and Aβ42 levels.

To determine whether AGEs accumulate in mitochondria in an Aβ-rich environment, mitochondrial and non-mitochondrial fractions were prepared from 4- and 12-month-old mAPP and nonTg mouse brains [15]. Mitochondrial purity was confirmed by enrichment of mitochondrial markers (TOM20 and VDAC) and the relative absence of Calnexin (endoplasmic reticulum, ER) and LAMP-1 (lysosomes) (Fig 2A & S1 Fig). We then measured AGEs levels in mitochondria and non-mitochondria from mAPP and nonTg mice. To examine the age-dependent accumulation of mitochondrial AGEs and their association with mitochondrial abnormalities, mice were analyzed at 4 months of age (at the start of cerebral Aβ accumulation) and 12 months (widespread cerebral Aβ deposit). Clearly, MG-AGEs and CML-AGEs were significantly elevated in cortical mitochondria of mAPP mice compared to nonTg mice in an age-dependent manner. Intriguingly, as early as 4 months of age, cerebral Aβ accumulation begins, mAPP mitochondria already showed significantly increased MG-AGEs and CML-AGEs. By 12 months, mitochondrial MG-AGEs and CML-AGEs reached 41.18 + 1.38 ng/mg protein and 104.46 + 1.48 ng/mg protein, respectively, ~ 2–3 fold higher than at 4-month mAPP mitochondrial AGEs (MG-AGEs: 14.43 + 1.49 and CML-AGEs: 57.94 + 2.34). Furthermore, mitochondrial MG-AGEs and CML-AGEs were significantly higher than non-mitochondrial fractions in both mAPP mice and nonTg mice (Fig 2B and 2C). Additionally, compared to 4-month-old non-Tg mice, the mitochondrial pool of MG-AGEs and CML-AGEs was significantly increased in 12-month-old mice, indicating that the accumulation of AGEs within mitochondria is age-dependent. (Fig 2B and 2C). These results suggest that AGEs and their precursor accumulate progressively in brain mitochondria in Aβ-rich AD mice. To further validate the presence of AGEs in mitochondria, immunogold electron microscope was performed in the intact mouse brain using a specific primary anti-AGE antibody, followed by a secondary antibody conjugated to gold particles as we described previously [53]. Apparently, AGEs immunogold particles were localized in mitochondria, including pre- and post-synaptic mitochondria in the mouse brain (Fig 2D). Pearson correlation analysis revealed that mitochondrial MG-AGEs and CML-AGEs were more strongly correlated with cerebral Aβ40 and Aβ42 (Fig 2E-L). These results suggest that mitochondrial accumulation of AGEs is progressive and pathological events that likely contribute to mitochondrial dysfunction in Aβ-rich AD brains.

### Effect of mitochondrial stress on AGE accumulation

**Fig 3:**
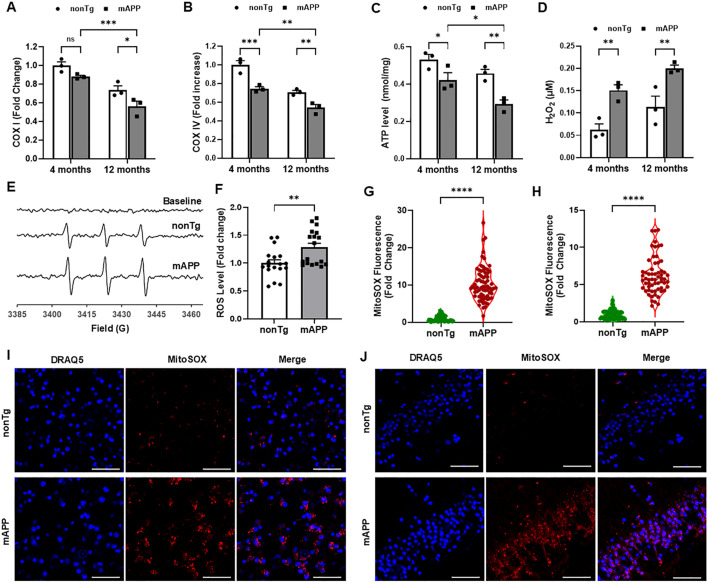
Association of mitochondrial dysfunction with AGEs accumulation. **(A-D)** Activity of mitochondrial complex I (COX I, **A**) and complex IV (COX IV, **B**) activity, ATP **(C)**, and H_2_O_2_ levels (**D**) in the indicated mice at 4 and 12 months of age. N = 3 mice per group, data were analyzed using 2-way ANOVA followed by the Fisher LSD test. **(E-F)** Representative spectra of EPR measurements of free radicals generated in the indicated mice **(E)**. The peak height in the spectrum indicates levels of ROS. Data are presented as a fold increase in mAPP mice relative to nonTg mice **(F)**. N = 18 images from each group of 6 mice, data were analyzed using an unpaired t-test. **(G-J)** Quantification of MitoSOX signal in cortex (**G**) and hippocampus (**H**) of indicated mice. For each group, 60-70 individual images obtained from 4-5 mice were quantified. Representative images of the cortex (**I**) and the hippocampus (**J**) are presented. Scale bar: 50 µm. *P < 0.05, **P < 0.01, ***P < 0.001, and ****P < 0.0001. ns = no significance.

We next examined whether mitochondrial AGE accumulation is associated with mitochondrial dysfunction. Mitochondrial respiratory function was assessed by measuring the activities of complexes I and IV, key enzymes of the respiratory chain. Human AD brain and AD mice show deficit in these enzyme activity [16,17]. As shown in Fig 3B, complex IV activity was reduced in mAPP mice as early as 4 months compared to nonTg mitochondria. By 12 months, activities of both complexes I and IV were further decreased by ~40% in mAPP mitochondria relative to 4-month nonTg mice (Fig 3A and 3B). Consistently, mitochondrial bioenergetics declined in mAPP mice, with reduced ATP levels observed at 4 months and further decreased at 12 months (Fig 3C). These findings indicate that mitochondrial respiration and energy metabolism are progressively impaired in Aβ-rich mitochondria.

Given that mitochondria are major sources of ROS generation and that impaired mitochondrial respiration increases ROS production, we measured hydrogen peroxidase (H_2_O_2_) levels in the brains of nonTg and mAPP mice. H_2_O_2_ levels were significantly elevated in mAPP mice at both 4 and 12 months compared to nonTg controls (Fig 3D). Intracellular ROS levels, assessed by highly specific electron paramagnetic resonance (EPR), were also markedly higher in mAPP cerebral cortex, as shown by the average height of EPR peaks (Fig 3E and 3F). Furthermore, mitochondrial ROS, measured using MitoSOX, which is selective for mitochondrial ROS, particularly superoxide, was significantly increased in both the cortex (Fig 3G and 3I) and hippocampus (Fig 3H and 3J) of mAPP mice. Together, AGE levels positively correlated with ROS and inversely correlated with mitochondrial function in Aβ-rich mAPP brains.

### Suppression of mitochondrial ROS mitigates AGE accumulation and restores mitochondrial respiration

**Fig 4:**
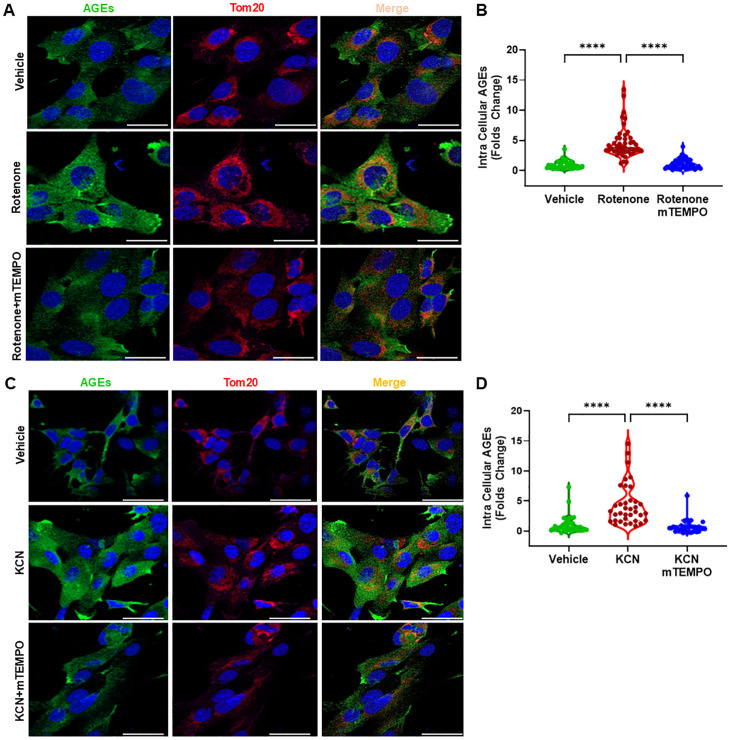
Effect of mitochondrial stress and ROS on the accumulation of AGEs in human neuronal cells. **(A)** Double immunostaining of SK-N-SH cells with AGEs (green) and TOM20 (red, mitochondrial marker) reveals elevation of colocalization of AGEs with TOM20 (merged yellow color) in rotenone-treated cells compared to vehicle-treated cells, which was significantly reduced in MitoTEMPO (mTEMPO) treatment. **(B)** Quantification of cellular AGEs in the indicated groups of cells as shown by fold change relative to vehicle-treated cells. **(C)** Double immunostaining of SK-N-SH cells with AGEs (green) and TOM20 (red) reveals elevation of AGEs in KCN-treated cells compared to vehicle-treated cells, which was significantly reduced in mTEMPO treatment. **(D)** Quantification of cellular AGEs in the indicated groups of cells as shown by fold change relative to vehicle-treated cells. N = 40-46 cells per group. Data was analyzed using one-way ANOVA followed by the Fisher LSD test. ****P < 0.0001. Scale bar: 50 µm.

Next, we addressed whether mitochondrial ROS contributes to AGEs accumulation. Disruption of mitochondrial respiration using rotenone, a complex I inhibitor, (Fig 4A and 4B) or KCN, a complex IV inhibitor, (Fig 4C and 4D) significantly increased cellular AGE levels. In contrast, scavenging mitochondrial ROS with MitoTEMPO, a mitochondria-targeted antioxidant, almost completely prevented the elevation of AGEs. Immunostaining with AGE and mitochondrial marker TOM20 confirmed the enhanced AGE accumulation, including mitochondrial localization, in rotenone- or KCN-treated cells (Fig 4A and 4C). Quantitative analysis showed a ~ 2–3-fold increase in AGEs compared to controls. Importantly, addition of MitoTEMPO to the cells completely blunted elevation of AGE accumulation. These results indicate that mitochondrial ROS drives both cellular and mitochondrial AGE accumulation.

**Fig 5:**
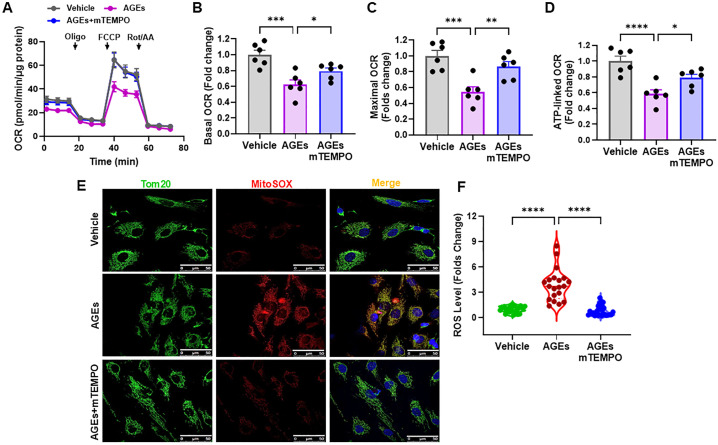
Effect of AGEs on mitochondrial respiration and bioenergetics. Representative OCR **(A)**, basal and maximal OCR **(B-C)**, and ATP-linked OCR of SK-N-SH cells with the treatment of AGEs (100 µg/mL) in the presence and absence of MitoTEMPO (2 µM, mTEMPO) **(D)**. N = 6, data were analyzed using one-way ANOVA followed by the Fisher LSD test. **(E-F)** Effect of AGEs on mitochondrial oxidative stress. **(E)** Representative double staining images of TOM20 with MitoSOX in cells treated with AGEs, AGEs + mTEMPO, and vehicle control. **(F)** Quantification of MitoSOX intensity in the indicated groups of cells. The result is shown as a fold change relative to vehicle control cells. N = 20-27 cells per group. Data was analyzed using one-way ANOVA followed by the Fisher LSD test. *P < 0.05, **P < 0.01, ***P < 0.001, and ****P < 0.0001. Scale bar: 50 µm.

To determine the direct effect of mitochondrial ROS on AGEs-induced mitochondrial dysfunction, human neuron-like cells (SK-N-SH) were exposed to AGEs in the presence or absence of MitoTEMPO. Mitochondrial function was assessed using the Agilent Seahorse XF platform to measure the capacity of mitochondrial respiration and ATP production in alive human neuron cells in real-time [47]. AGE treatment significantly reduced basal and maximal respiration, as well as mitochondrial ATP production (Fig 5A-D). Consistently, mitochondrial ROS levels, measured by MitoSox, were markedly increased in AGE-treated cells and were effectively suppressed by MitoTEMPO (Fig 5E and 5F). These results identify mitochondrial ROS as a key mediator of AGEs-induced mitochondria dysfunction. Its inhibition protects against mitochondrial injury and oxidative stress.

## Discussion

Although AGEs are formed during normal aging, their accumulation is accelerated in neurodegenerative diseases, including AD, Parkinson’s, and Huntington’s diseases [4,8,9,48–50]. AGE-modified proteins form crosslinks that promote aggregation and insolubility. They also serve as a continuous source of reactive oxygen species. In particular, AGE-modified Aβ and tau exacerbate oxidative stress and neuronal dysfunction [8–10]. Elevated AGE levels have been reported in the cortical neurons of older adults and are positively correlated with cognitive decline, suggesting that excessive AGE accumulation could play a causative role and/or promote cellular perturbation and modify disease processes. Understanding the causes and consequences of AGE formation and accumulation is therefore critical for elucidating AD pathogenesis. Our previous study demonstrated an association of age-related AGE accumulation with mitochondrial dysfunction and oxidative stress. However, whether mitochondrial ROS directly drives AGE accumulation remains unclear. Given that mitochondrial dysfunction is a hallmark of brain aging and AD, and that Aβ can induce mitochondrial impairment and excessive ROS production, we propose that Aβ-mediated mitochondrial perturbation accelerates AGE formation and accumulation.

The present study suggests that mitochondrial dysfunction–mediated oxidative stress contributes to AGE formation and accumulation, providing a mechanistic link between mitochondrial stress, toxic metabolites, and AD pathogenesis. First, AGE levels were significantly elevated not only in the cerebral cortex but also in cortical mitochondria of Aβ-overexpressing AD mice. AGE accumulation occurred in an age-dependent manner, with increases detectable as early as 4 months prior to substantial amyloid deposition, and showed a positive correlation with cerebral Aβ levels. Second, blockade of mitochondrial oxidative stress almost completely suppressed AGE accumulation, indicating that mitochondrial ROS is a key driver of AGE formation. Functionally, AGE levels in both the brain and mitochondria were inversely correlated with mitochondrial function. AGEs can also directly impair mitochondrial activity and ATP production. Importantly, these detrimental effects were reversed by a mitochondria-targeted antioxidant. Together, these findings suggest a synergistic interaction in which Aβ-induced mitochondrial dysfunction promotes AGE accumulation, further exacerbating mitochondrial and neuronal impairment in the AD brain.

Although Aβ disrupts mitochondrial function and causes oxidative stress [51], its interaction with AGEs with mitochondria significantly enhances the accumulation and production of mitochondrial ROS [52]. Excessive ROS further exacerbates oxidative damage and mitochondrial malfunction, including the collapse of the mitochondrial membrane potential [15,53]. This effect has been observed in Aβ-produced AD mice, which show increased accumulation of AGEs and mitochondrial ROS, along with greater mitochondrial polarization. Aβ may also promote the accumulation of AGEs in the human brain, contributing to the formation of neurofibrillary tangles and senile plaques during aging. In elderly individuals, AGEs are elevated in cortical neurons and cerebral vessels and are associated with the severity of cognitive impairment. Older adults are at higher risk of developing neurological disorders, including AD, which is positively correlated with the extent of AGE deposition and the upregulation of the AGE receptor RAGE [54].

Emerging evidence indicates that impaired mitochondrial function and oxidative stress, driven by excessive ROS production, are implicated in the onset and progression of neurodegenerative diseases. Mitochondrial ROS contribute to synaptic dysfunction and cognitive decline during aging and in AD [6,7,15–17,55]. Mitochondria-targeted antioxidants have been shown to attenuate Aβ-, AGE-, and aging-induced mitochondrial oxidative stress, restore mitochondrial and synaptic function, and improve learning and memory in AD mouse model. Furthermore, administration of MitoTEMPO to aging mice significantly reduces cerebral AGE accumulation, reverses mitochondrial dysfunction, and suppresses ROS production [6]. However, the direct effects of mitochondria-targeted antioxidant on AGE accumulation in Aβ/AD mouse model with amyloid pathology remain to be determined. Additionally, assessing the impact of other antioxidants, including natural product-derived compounds, on AGE metabolism may help advance therapeutic strategies for age-related dementia and cognitive decline [56]. Mitochondrial ROS-mediated signal transduction pathways, including MAP kinase pathways [17,42,57,58] and protein kinase A (PKA)/cAMP regulatory-element-binding (CREB) [59] pathways play important roles in oxidative stress signaling cascade that lead to mitochondrial and synaptic injury in brain aging and AD pathogenesis. Therefore, studying how mitochondrial signaling contributes to AGE formation and accumulation may help define the crosstalk between mitochondria stress and AGE metabolism.

Notably, the relationship between mitochondrial dysfunction and AGE formation is complex, as these processes mutually reinforce one another through a feedback loop. Furthermore, findings derived from in vitro systems and animal models may not fully recapitulate the complexity of human disease. The development of biomarkers capable of simultaneously monitoring mitochondrial health and AGE levels may offer novel avenues for therapeutic intervention in aging and age-related diseases.

## Conclusion

We propose that aging and the accumulation of toxic metabolites, such as Aβ, trigger mitochondrial stress and ROS production, thereby contributing to AGE accumulation. AGEs act as “progression factors” in AD, amplifying preexisting AD pathology and mitochondrial failure. This feed-forward cycle of mitochondrial impairment and ROS overproduction accelerates AGEs formation and accumulation, ultimately promoting neurodegeneration and AD progression. AGE-mediated ROS signaling may further exacerbate mitochondrial dysfunction, and amyloid pathology, synaptic injury, and cognitive decline. These findings suggest that targeting AGE or ROS formation, by preserving mitochondrial function and integrity, could be a promising strategy to slow AD progression and age-related neurodegeneration.

## Supporting information

S1 Fig Immunoblot image data as the initial source of Fig 2A. Immunoblotting of cortical homogenates and mitochondria fraction for lysosome (Lamp-1), endoplasmic reticular (ER, Calnexin), β-actin, mitochondrial markers (VDAC and TOM20).
(TIF)
