# Supplementary figures and images for: Mitochondrial oxidative stress promotes the accumulation of advanced glycation end products
Source: PLoS One. 2026 Jun 23;21(6):e0352355. doi: 10.1371/journal.pone.0352355 (PMC13289938; doi:10.1371/journal.pone.0352355)

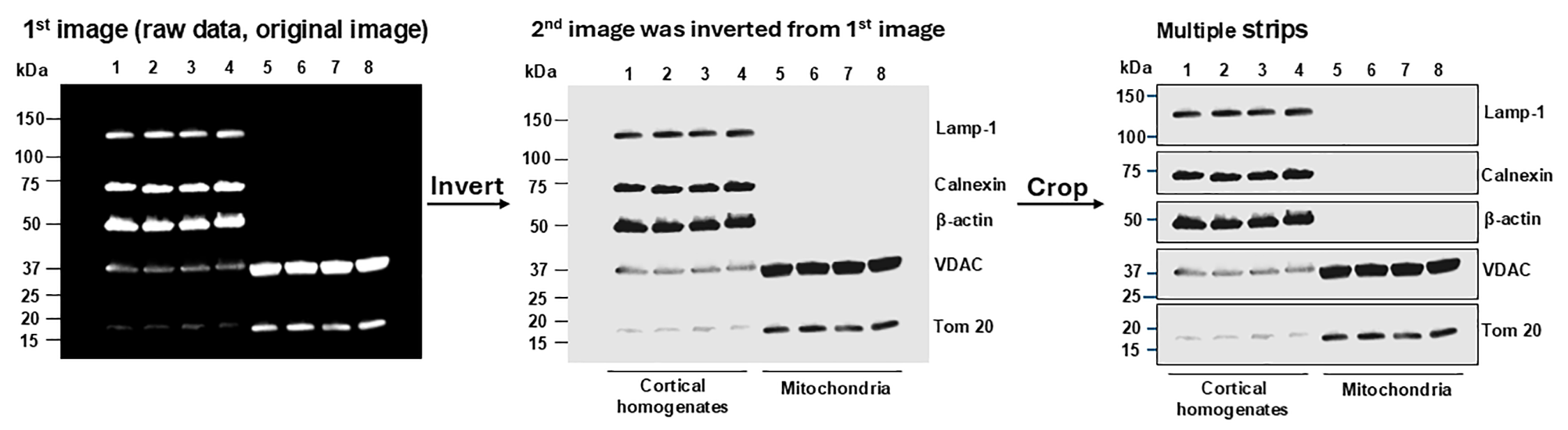

Supplement: S1 Fig — Immunoblotting of cortical homogenates and mitochondria fraction for lysosome (Lamp-1), endoplasmic reticular (ER, Calnexin), β-actin, mitochondrial markers (VDAC and TOM20). (TIF) [file pone.0352355.s001.tif]
